# The Causes and Prevention of Infantile Diarrhœal Diseases (continued)

**Published:** 1887-07

**Authors:** F. R. Campbell

**Affiliations:** Lecturer on Hygiene, Niagara University, Sanitary Inspector of the Board of Health, Buffalo, N. Y.; 31 Franklin street


THE BUFFALO MEDICAL AND SURGICAL JOURNAL

VOL. XXVII. JULY, 1887. NO. 12

### Original Communications.

### *THE CAUSES AND PREVENTION OF INFANTILE DIARRHŒAL DISEASES.*

BY F. R. CAMPBELL, A. M., M. D.

Lecturer on Hygiene, Niagara University,

Sanitary Inspector of the Board of Health, Buffalo, N. Y.

(*Concluded.*)

### IV. SANITARY CAUSES.

Summer diarrhœa is classified by sanitarians as a filth disease. It is common and fatal in cities, but rare in the rural districts. It is most prevalent in filthy localities, where the soil is contaminated and the sewerage and drainage are imperfect. In Buffalo it is most fatal in the thickly settled portion of the Fifth Ward, where there is a shallow clay soil poorly drained. There is least of this affection in the Ninth Ward, which has a sandy soil, well drained and occupied by a wealthier class of people. Many of the streets in the Fifth Ward occupied by Poles are without sewers, pavements, or public water, the houses are overcrowded, the occupants filthy; garbage collects in the yards and is often thrown into the street. Many of the families occupy but one or two rooms. Soiled diapers are dried by the fire where the family diet is prepared. The children are seldom bathed, the adults never. When a child becomes ill a physician is seldom called until a death certificate is needed. It is not surprising then that the mortality from diarrhœal affections in these localities is nearly 100 per cent. above the average for the entire city.

Just how filth produces diarrhœa is not well known, for the same factors seem to vary in their effects upon different individuals. When the explorers Stanley and Dr. Livingstone were traveling together in Africa it was observed that the fetid miasms from swamps and marshes would cause fever in Stanley and dysentery in Dr. Livingstone.

Many hypotheses have been offered to explain how the emanations from decomposing matter may cause diarrhœal disease, but as yet nothing positive have been demonstrated. We will mention—

(1) *The Chemical Hypothesis.*—Dr. D. F. Lincoln maintains that the sulphuretted hydrogen which is always evolved during process of decomposition of filth is converted into sulphuric acid gas in the air, and unites with sodium and ammonia bases to form purgative salts, sulphates of soda and ammonia. The continued inhalation of these gases and salts, he claims, will produce diarrhœa. In many persons the inhalation of sulphuretted hydrogen will produce digestive disturbances, malaise and diarrhœa.

(2) *The Zymotic Hypothesis.*—While the gaseous constituents of foul air may have some influence in the production of diarrhœa, there are undoubtedly other substances of far greater importance. The greatest number of living germs is found in the air of filthy localities. The zymoses are supposed by the majority of pathologists to be living ferments. Burkart in 1873 investigated a form of diarrhœa which he denominated intestinal mycosis. This affection was supposed to be caused by taking diseased meat into the alimentary canal. A fungus was discovered in the meat and one which was identical with it growing in the intestines, even penetrating the lining epithelial cells. Walder in 1878, Bollenger in 1881, and Klein in 1885 studied the diarrhœal diseases of infants, and found that numerous micro-organisms, micrococci, vibriones and bacilli were always present in the intestines in these affections, but as yet no specific germ has been demonstrated.

(3) *The Ptomaine Hypothesis.*—Ptomaines, the alkaloidal substances formed by putrefaction, are now attracting great interest in their relation to disease. Le Bon sent a communication to the French Academy of Medicine giving the results of his investigation of the cause of cholera and choleriform diarrhœa in India. The sacred ponds in India are often pools of stagnant water where the pious devotees of Brahma make their ablutions. Le Bon observed that Asiatic cholera and diarrhœa prevailed in the vicinity of one of these ponds. A chemical examination of the air and water revealed, besides the usual micro-organisms and gases, a ptomaine which he believed to be the cause of the diarrhœal affection. "When air containing this ptomaine was inhaled for a long period the subject would be affected with true cholera, often of a fatal character, but when inhaled for a short period only, a choleriform diarrhœa was produced." In other words the character of the disease produced depended upon the dose of ptomaines taken into the system.

### V. DIETETIC CAUSES.

Dujardin-Beaumetz has said that "the new-born infant is a digestive tube served by organs, and these organs are fitted to assimilate but one kind of food—the mother's milk." When food that cannot be digested is taken into the alimentary canal it undergoes putrefactive changes, and poisonous and irritating substances are formed, which induce inflammatory changes and diarrhœa. Probably the greatest discovery in physiology which has been made in this century is that of Armand Gautier, who has demonstrated beyond peradventure that the cells of animals as well as plants secrete alkaloids, substances which he has called *leucomaines.* These alkaloids are almost identical with those formed in plants; some act like strychnine, others like muscarine. Indeed Brieger has succeeded in transforming neurine, a harmless animal substance, into muscarine, a very poisonous alkaloid, by leaving it exposed in an aqueous solution to the air. A plant, when the atmospheric conditions and the character of the soil are not adapted to its growth, will form abnormal alkaloids, become diseased and die. It is quite probable that the cells of animals will act in the same manner, forming abnormal alkaloids and producing an auto-intoxication when the food and surrounding atmosphere are unsuitable, especially in the case of infants, whose organisms have not yet acquired the power of resisting untoward influences. Ptomaines are always present in the intestines. Tanret has obtained them by placing alkaline salts in contact with peptones. Brieger has found them where meat has been digested by gastric juice. When the intestinal membranes are in a normal condition and when the food is properly digested, the alkaloids are not absorbed into the blood, or are not of a poisonous character. But when putrefaction instead of digestion takes place in the alimentary canal, abnormal alkaloids are formed which, when absorbed through the intestinal membranes, produce a toxæmic diarrhœa* It is, moreover, probable that the congested intestinal mucosa will absorb substances which would not be taken up by the healthy membrane, just as the mucous lining of the bladder, when inflamed, will absorb readily what could not possibly be taken up in health. May we not explain the convulsions of infants so commonly observed in hot weather, by the absorption into the blood of an excessive quantity of these leucomaines, which resemble strychnine in their action? And is it not quite probable that some of the forms of diarrhœa — the choleraic, for example — are simply manifestations of an effort of nature to eliminate the poisons from the system?

Even the mother's milk will at times produce diarrhœa in

*At the Seance of the Paris Academy of Medicine, May 17, 1887, Hayem presented a communication on the treatment of dyspepsia of infants and the green diarrhœa of children. Having observed that the arrival of a child affected with green diarrhœa was the signal of an epidemic of this affection among the children in the crêche, Hayem suspected that the disease was of a contagious character and ordered a thorough disinfection of the stools and soiled clothing. As a result the epidemic immediately began to disappear. In connection with Dr. Lesage he has demonstrated that the green color of the stools is produced by a special bacillus which secretes a green substance during its development in the intestine. Hayem finds lactic acid the best germicide for the destruction of this bacillus.

the infant. When the mother is in ill health, when she becomes pregnant, and sometimes when the catamenia re-appear, changes take place in the milk which render it unfit for food. In some of these cases colostrum cells are detected, which account for the purgative action; in other cases nothing can be discovered to explain the effects observed. If nursing women take an excess of nitrogenous food, diarrhœa will be produced in the infant, especially in hot weather. In these cases there is an excess of casein in the milk. This casein is in part converted into peptones in the stomach, and the peptone alkaloids may be formed in too great quantities to be properly disposed of without inducing diarrhœa. When children take an excess of nitrogenous food it cannot all be disposed of in the construction of tissues, and an attack of diarrhœa and vomiting, with febrile disturbance, is produced to destroy and eliminate the waste products. To use the words of Fothergill, "There is a grand cleaning up, accompanied by a bonfire." Overfeeding, then, may be given as one of the causes of diarrhœa, even though nothing but mother's milk is given.

When infants obtain their milk from wet nurses the mortality is nearly double that of children nursed by their mothers One of the best things which can be said of the good Queen of England is that she nursed every one of her ten children at her own breast. In some countries wet-nursing is the "trade," as Monot calls it, of thousands of women. But few of the well-to-do women of Paris nurse their own children. The wet nurses come from the country about the metropolis. During twelve years, in the village of Montsauche, 3,950 women were confined, and 2,710—nearly 70 per cent.—became wet nurses, 1,260 bringing children to the country and 1,450 going to Paris. During this period the mortality of the children of Montsauche, under one year of age, was 33 per cent. During sixteen months, in 1870 and 1871, when Paris was besieged, the women could not procure their infant charges from the city, and the mortality immediately dropped to 17 per cent.—about one-half. This statement needs no comments to show the immense superiority of maternal lactation. In Scotland wet nurses are almost unknown, and the infant mortality is the lowest in Europe — 11 per cent. Even in Ireland, that land of famine, filth and fights, the infant mortality is but 14 per cent., a fact largely due to the universality of maternal lactation. Nature seems to adapt the milk of the mother to the wants of the child according to its age, the milk of another woman being liable to cause indigestion and diarrhœa.

In our country, as in Scotland, the wet-nurse trade is almost unknown. But we have a far more reprehensible substitute—the nursing bottle. When the foundlings of New York were sent to the alms-house, one that attained the age of seven months was looked upon as a curiosity. Of the bottle-fed infants in the "baby farms" near Paris, 71 per cent. die during the first year. In New York more than one-half of the dry-nursed infants, under six months of age at the beginning of the hot season, die of diarrhœa before the summer is passed, and in the "baby farms" the mortality is even greater.

In the Edward Street Infant Asylum, of Buffalo, the Sister Superior informed me that if a bottle-fed baby lives to be three months old, even in winter, they think they have done remarkably well in preserving it to such a mature age, and in the summer to bring a foundling there is only a step before taking it to the cemetery. Furthermore she stated that 75 per cent. of the foundlings taken there died whether they were wet nursed or "brought up on a bottle." So great has been the mortality and so discouraging have been the results, that she now refuses, as far as possible, to take foundlings in the institution. Last year more than three-fourths of the babies had their mothers with them, and yet the mortality was over 40 per cent. In reply to my question as to the nature of the diseases of which they died, she said that some seemed to waste away, but in summer they have diarrhœa and inflammation of the bowels. Those that are not nursed by their mothers, she said, have "one cow's milk from the country." In view of these facts, we must grant that the great fame acquired by Mrs. Joseph Gargery for having brought Pip up by hand, was exceedingly well deserved. In countries where dry-nursing is rare, the second summer of the child's life is considered most dangerous to its health, a fallacy which has been brought to our country by many foreigners.

A large share of the mortality from diarrhœal affections must be attributed to the use of unhealthy milk. In Holland, she asses are driven about the streets and milked before the houses where bottle-fed infants are found. It is said that the mortality among children thus nourished is exceedingly low. Competent authorities state that next to the milk of the mother, that of the ass is the best adapted to the infantile digestive organs. But asses' milk is not obtainable here, and even in Paris it brings $1.50 per quart. We are accordingly obliged in cities to content ourselves with milk which is supposed to have been derived from the cow. It is absolutely impossible to obtain fresh milk, and we are extremely fortunate if we can procure that which has not undergone partial decomposition.

Cows' milk will produce diarrhœa in children under the following circumstances:

(1) Where the milk contains colostrum. This substance is normally present in milk for a number of days after parturition; it is also secreted when the udder is inflamed, and when the cow is in poor health.

(2) When the diet of the cow is improper. It is a well-known fact that poisonous substances taken in with the food will contaminate the milk. A large number of children in Rome, fed on goats' milk, were poisoned, the symptoms being vomiting and diarrhœa. It was found that the food of the goats contained colchicum, and the alkaloid of this plant was detected in the milk. An enormous quantity of refuse from starch and glucose factories, and from breweries and distilleries, is fed to the cows in and near cities. A thorough chemical examination of these waste products has never yet been made. There are many organic chemical compounds which have not yet been isolated in the laboratory, and it may be some of these which pass into the milk and cause the dyspepsia and diarrhœa of infants. It is a well-known fact that the milk of women and grass-fed cows is alkaline, while that of stall-fed cows, when starch refuse is used, is acid, and ferments rapidly. Fermented milk will almost invariably cause gastro-intestinal disturbances in infants.

(3) The milk may become contaminated after it has left the cow with substances which will produce diarrhœa. Milk will absorb gases and sustain the life of micro-organisms. Lister considers it the best culture fluid in existence. Nature never intended that milk should be splashed in wagons about our streets in hot weather, poured from can to pail, from pail to pitcher, and fed to the baby perhaps 24 hours after it has left its native fountain. Such milk is of necessity in a state of decomposition, and decomposition means the formation of chemical substances which are in many cases poisonous, a fact which has been demonstrated by Dr. Vaughan of Mich., who has succeeded in isolating tyrotoxicon from samples of poisonous milk and cheese.

Much of the milk is now transported in air-tight bottles and people erroneously believe that fermentation is thus arrested. This is not the case, however, if the milk has not been boiled before being placed in the cans. If the milk is placed in the cans before it is cooled, and the morning milk is seldom cooled, fermentation goes on in the can and poisons are formed. Selmi has shown that the ptomaines formed when oxygen is deficient, as it must be in bottled milk, are far more poisonous than the ptomaines formed when oxygen is abundant.

Lastly, the ingestion of food not adapted to the age of the child will cause diarrhœa. It is not until the child has cut its first teeth that the glycogenic ferments are found in the salivary and pancreatic secretions, and until these ferments are formed, starches cannot be digested, but undergo decomposition, producing what Dujardin-Beaumetz calls putrid dyspepsia, an affection always attended by diarrhœa. If carbohydrate food is given at all before the first dentition, it should be in the form of maltose or grape sugar.

Moreover, the gastric juice in early life is deficient in pepsin.

For this reason nitrogenous food, other than milk, should not be given in large quantities.

Fruit in cities is a dangerous article of diet for children. It is picked when green to avoid early decay, and often lies in markets and groceries surrounded by decaying vegetables, and is sometimes liable to contamination from sewers, as is the case in our Elk Street Market. In the country enormous quantities of fruit are eaten by the children, and yet it seldom does harm unless it is unripe or matured too early on account of disease. But stale fruit contains poisons which will almost uniformly produce diarrhœa.

### PREVENTIVE MEASURES.

From a review of the above conditions which predispose to diarrhœal diseases in children, we can easily deduce methods for their prevention. As the liability to these diseases varies inversely with the age, especial care must be exercised in the management of early infancy. And it will also be observed that male children will require more careful attention than female. By increasing the comforts and intelligence of the working classes, by removing their vices, we will greatly reduce their infant mortality.

Foundlings and illegitimate children should not be kept in asylums or alms-houses in the city, but should be sent to some establishment in the country, like the Thomas Wilson Sanitarium for children of Baltimore, where the best care and suitable food can be provided.

In hot weather the children should be kept out of the heat, and sudden chills should be avoided. Damp night air is especially to be feared. The bath should be given during the heat of the day, and not in the morning or evening. A flannel bandage should be worn over the abdomen to prevent sudden chills. All filth and dirt about the house or premises, such as foul garbage barrels, stinking privy vaults, or foul sewer receivers, should be looked upon as poisons liable to produce diarrhœa and death.

The municipal authorities should see that good water, adequate drainage and sewerage, and clean streets are provided in all sections of cities, especially in summer. Water should be given to the baby frequently during the hot season. Mothers should, as far as possible, nurse their own children during the first nine or twelve months of life. Children should never be weaned just before or during the hot season; that is, not later than May or before October.

When cows' milk is used it should be boiled and cooled before using. In this way not only are micro-organisms and poisons largely destroyed and fermentation arrested, but the milk is rendered more digestible. The milk should, if possible, in summer be boiled and bottled at the dairy. A careful surveillance of the milk supply should be exercised by the health authorities in all cities, as the evil too often exists in the food supply and condition of the cows.

During the first six months of life the diet should be milk, and milk alone, then a small amount of starchy food may be added, while meat should not be given before the child is three years of age. At this age also, fruit may be added to the diet, but uncooked fruit should be avoided in cities during the hot season.

Lastly, when cases of cholera infantum or green diarrhœa make their appearance in any locality, the physician should insist upon the careful disinfection of the alvine discharges and soiled clothing with lactic acid or strong solutions (1 per cent.) of corrosive sublimate.

31 Franklin street, Buffalo, N. Y.

